# Development of a Specific Monoclonal Antibody-Based ELISA to Measure the Artemether Content of Antimalarial Drugs

**DOI:** 10.1371/journal.pone.0079154

**Published:** 2013-11-13

**Authors:** Suqin Guo, Yongliang Cui, Lishan He, Liang Zhang, Zhen Cao, Wei Zhang, Rui Zhang, Guiyu Tan, Baomin Wang, Liwang Cui

**Affiliations:** 1 College of Agronomy and Biotechnology, China Agricultural University, Beijing, China; 2 Department of Entomology, Pennsylvania State University, University Park, Pennsylvania, United States of America; London School of Hygiene and Tropical Medicine, United Kingdom

## Abstract

Artemether is one of the artemisinin derivatives that are active ingredients in antimalarial drugs. Counterfeit and substandard antimalarial drugs have become a serious problem, which demands reliable analytical tools and implementation of strict regulation of drug quality. Structural similarity among artemisinin analogs is a challenge to develop immunoassays that are specific to artemisinin derivatives. To produce specific antibodies to artemether, we used microbial fermentation of artemether to obtain 9-hydroxyartemether, which was subsequently used to prepare a 9-O-succinylartemether hapten for conjugation with ovalbumin as the immunogen. A monoclonal antibody (mAb), designated as 2G12E1, was produced with high specificity to artemether. 2G12E1 showed low cross reactivities to dihydroartemisinin, artemisinin, artesunate and other major antimalarial drugs. An indirect competitive enzyme linked immunosorbent assay (icELISA) developed showed a concentration causing 50% of inhibition for artemether as 3.7 ng mL^−1^ and a working range of 0.7–19 ng mL^−1^. The icELISA was applied for determination of artemether content in different commercial drugs and the results were comparable to those determined by high-performance liquid chromatography analysis. In comparison with reported broad cross activity of anti-artemisinin mAbs, the most notable advantage of the 2G12E1-based ELISA is its high specificity to artemether only.

## Introduction

Despite intensive international efforts, malaria still affects ∼5% of the world’s population [1]. To deal with the spread of multidrug resistance malaria parasites, most falciparum-endemic countries have switched to artemisinin-based combination therapies (ACTs) for treating *Plasmodium falciparum* [2]. Among a number of ACTs, artemether-lumefantrine (Coartem®) is a fixed-dose oral combination for treating uncomplicated falciparum malaria in adults and children [3]. Currently, the artemisinin-based antimalarial drugs mainly include artesunate, dihydroartemisinin and artemether. However, the circulation of counterfeit and substandard artemisinin-based antimalarial drugs greatly threatens the malaria control campaign. This problem demands implementation of strict regulation of drug quality by the regulatory authorities of these affected nations. For this purpose, fast and reliable methods of artemisinin derivatives detection and quantitation are needed.

Currently, a number of analytical methods have been developed for detecting and quantifying artemisinin and its derivatives, including high-performance liquid chromatography (HPLC) [4], liquid chromatography-mass spectrometry (LC-MS) [5], ultraviolet spectroscopy [6], thin layer chromatography (TLC) [7], [8], generic micellar electrokinetic chromatography [9], and gas chromatography-mass spectrometry (GC-MS) [10]. These instrumental methods, however, require expensive instruments and highly qualified personnel, and are often laborious and time-consuming. TLC method is simple to use, rapid and cost-effective, but it often uses toxic and flammable reagents and is not quantitative. In this regard, immunoassays such as the enzyme-linked immunosorbent assay (ELISA) have been developed for quantifying artemisinin and its derivatives [11]–[14]. Though very sensitive and simple to perform, immunoassays often have the limitations from cross reactivity to molecules with similar structures. Artemisinin and its derivatives used in antimalarial drugs differ at position 12 (numbered according to Abourashed EA [15]) of their molecular structures (e.g., O-H, βO-CH_3_, βO-CH_2_CH_3_, and αO-COCH_2_CH_2_COOH). To generate antibodies against a small molecule such as artemisinin, the primary immunogen needs to be conjugated to a carrier protein such as ovalbumin (OVA) or bovine serum albumin (BSA). All antibodies against artemisinin and its derivatives reported in the literature were produced with immunogens of which the carrier protein was conjugated on position 12 of artemisinin derivatives because of the presence of an active side chain in artesunate. Antibodies generated in this way cross-react with all artemisinin derivatives. For example, a monoclonal antibody 3H2 obtained earlier in our laboratory displayed significant cross reactivity to artemisinin, dihydroartemisinin, and artesunate, and to a lesser degree, artemether [16].

**Figure 1:**
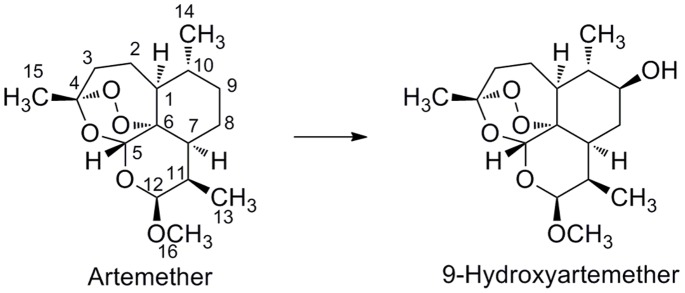
Microbial transformation of artemether.

To develop specific monoclonal antibodies for different artemisinin derivatives, it is necessary to conjugate the artemisinin derivatives at positions opposite to position 12 (preferably at position 9) so that the peroxide group and derived groups at position 12 are fully exposed (Fig. 1). However, artemisinin and its derivatives having an active group at position 9 are difficult to obtain by chemical synthesis. There are reports that microbial fermentations of artemisinin and its derivatives could produce structurally complex products, among which molecules containing active groups at position 9 could be isolated [15]. These molecules have been isolated in analytical quantities and may not be sufficient for developing an immunoassay-based method. In this study, we employed a microbial fermentation procedure to obtain sufficient quantities of 9-hydroxyartemether, which was used to obtain a monoclonal antibody with high specificity for artemether. The assay was then used to quantify artemether content in commercial antimalarial drugs.

## Methods

### Animal Treatment

Female Balb/c mice were purchased from the Laboratory Animal Center of the Institute of Genetics (Beijing, China). This study was performed strictly according to the standards described in the “Guide for the Care and Use of Laboratory Animals” (National Research Council Commission on Life Sciences, 1996 edition). All animal treatment procedures were approved by the Animal Care Committee of China Agricultural University (CAU) and all efforts were made to minimize suffering. Mice were housed under controlled temperature (22±2°C) and lighting (12 h light/12 h darkness) with food and water ad libitum in air–conditioned rooms. All experimental mice were killed by cervical dislocation.

### Materials

*Cunninghamella elegans* (ATCC 9245) was from American Type Culture Collection. Artemisinin, artesunate, dihydroartemisinin, and artemether were purchased from the National Institute for the Control of Pharmaceutical and Biological Products (Beijing, China). Quinine and primaquine phosphate were purchased from J&K Chemical (Beijing, China). Chloroquine diphosphate salt, pyrimethamine and lumefantrine were purchased from Sigma (St Louis, MO, USA). Artemether injection (Kunming Pharma. Corp.) and artemether soft capsules (Chongqing Holley Healthpro Pharmaceutical CO., Ltd.) were purchased from Beijing International Travel Healthcare Center. Coartem 20/120 (Beijing Novartis Pharma Ltd.) was purchased from Addis Ababa, Ethiopia. Co-Falcinum (Cipla, Ltd.) was purchased from Kenya. 1-(3-Dimethyl amine propyl)-3- ethylcarbodiimide (EDC), *N*-hydroxysuccinimide (NHS), succinic anhydride, 4-dimethylamino-pyridine (DMAP), BSA, OVA, polyethylene glycol 2000, dimethyl sulfoxide (DMSO), hypoxanthine, aminopterin, and thymidine (HAT), hypoxanthine and thymidine (HT) medium supplements, penicillin, streptomycin, l-glutamine, horseradish-peroxidase-labeled goat anti-mouse IgG, complete and incomplete Freund’s adjuvant, and o-phenylenediamine (OPD) were purchased from Sigma. Cell culture medium (Dulbecco’s modified Eagle’s medium, DMEM) and fetal bovine serum (FBS) were obtained from Gibco BRL (PaisLey, Scotland). All other chemicals and organic solvents used were of analytical grade and purchased from Sinopharm Chemical Reagent (Beijing, China).

### Preparation of 9-hydroxyartemether

9-Hydroxyartemether was obtained by microbial transformation of artemether by *C. elegans* (ATCC 9245) (Fig. 1) as described previously [15]. *C. elegans* was grown at 27°C in 20 500-mL culture flasks with each flask containing 200 mL of medium. A total of 1000 mg of artemether (in 10 mL of ethanol) was evenly distributed among the 24 h old stage II cultures. After 4 days, the incubation mixtures were pooled and filtered to remove the cells and the filtrate (4 L) was extracted three times with ethyl acetate. The combined extracts were dried over anhydrous sodium sulfate and evaporated to dryness at 35°C under reduced pressure to obtain a brown residue. The residue was purified with a silica gel column (30 g, 25 cm) using a hexane-ethyl acetate (10/1, v/v) mixture as the eluting system to afford 9-hydroxyartemether as white crystalline solid. MS m/z calcd for C_16_H_27_O_6_ [M+Na]^+^337.16, found 336.83; ^1^H-NMR (CDCl_3_, 300 MHz): δ 5.42 (1 H, s), 4.68 (1 H, d), 3.41 (3 H, s), 3.10 (1 H, m), 2.60 (1 H, m), 2.36 (1 H, m), 1.9–2.1 (1 H, m), 1.59 (1 H, m), 1.5–1.9 (2 H, m), 1.44 (3 H, s), 1.2–1.4 (1 H, m), 1.05 (3 H, d), 0.90 (3 H, d); ^13^C-NMR (CDCl_3_,75 MHz): δ 104.1, 103.2, 87.4, 80.3, 74.2, 56.0, 50.0, 44.2, 42.0, 36.3, 33.6, 30.6, 26.1, 24.6, 15.4, 12.9.

### Preparation of the Hapten 9-O-succinylartemether

**Figure 2:**
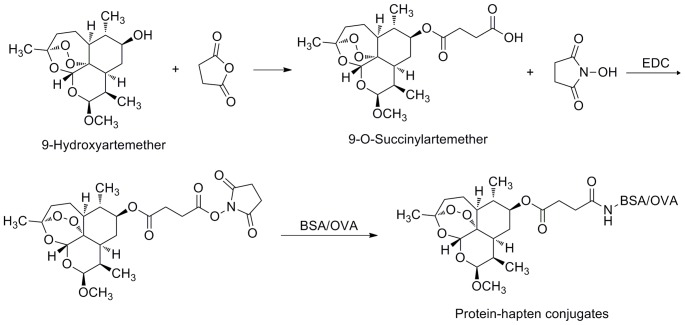
Preparation of artemether hapten and protein-hapten conjugate.

Succinic anhydride (89.8 mg) was added to 146 mg of 9-hydroxyartemether in 25 ml anhydrous CH_2_Cl_2_ and stirred at 4°C. DMAP (49.7 mg) was added subsequently and stirred at 0–5°C for 30 min. The reaction was warmed to room temperature naturally and stirred for 1 h. Chemical synthesis was monitored by TLC developed with ethyl acetate/petroleum ether (1/1, v/v). The reaction solution was poured into 25 mL water, and the mixture adjusted to pH 3.0 using 10% hydrochloric acid. The solution was washed with water (3×25 mL), dried over anhydrous sodium sulfate, and concentrated under reduced pressure (Fig. 2). The product was recrystallized from hexane-ethyl acetate as white crystalline solid. MS m/z calcd for C_16_H_27_O_6_ [M+Na]^+^337.16, found 336.83; ^1^H-NMR (CDCl_3_, 300 MHz): δ 5.42 (1 H, s), 4.68 (1 H, d), 3.41 (3 H, s), 3.10 (1 H, m), 2.60 (1 H, m), 2.36 (1 H, m), 1.9–2.1 (1 H, m), 1.59 (1 H, m), 1.5–1.9 (2 H, m), 1.44 (3 H, s), 1.2–1.4 (1 H, m), 1.05 (3 H, d), 0.90 (3 H, d); ^13^C-NMR (CDCl_3_,75 MHz): δ 104.1, 103.2, 87.4, 80.3, 74.2, 56.0, 50.0, 44.2, 42.0, 36.3, 33.6, 30.6, 26.1, 24.6, 15.4, 12.9.

### Preparation of Immunogen and Coating Antigen

The resulting hapten 9-O-succinylartemether was conjugated to OVA and BSA as immunogen and coating antigen, respectively (Fig. 2). Briefly, 4.17 mg EDC and 2.5 mg NHS were added to 15 mg of 9-O-succinylartemether in 1 mL of DMSO. The solution was stirred overnight at 4°C. The reaction mixture was added dropwise to 42.45 mg of BSA or 26.87 mg of OVA dissolved in 5 mL of 0.01 M phosphate buffered saline (PBS) and stirred overnight at 4°C. The mixture was dialyzed against 2 L of 0.01 M PBS (pH 7.5) containing 0.15 M NaCl for 3 days with two changes per day, then lyophilized and stored at −20°C.

### Preparation of mAb against Artemether

The mAb against artemether was prepared according to the procedures described previously [16], [17]. Balb/c mice were immunized with 100 µg of the immunogen using an equal volume of complete Freund’s adjuvant. Mice were subsequently injected twice with the immunogen emulsified with incomplete Freund’s adjuvant at 2-week intervals. The best-performing mouse was boosted with 100 µg of immunogen in 100 µL PBS 3 days before fusion.

Spleen cells collected from the mouse were fused with murine SP2/0 myeloma cells which were grown in complete medium (DMEM supplemented with 15% FBS, 0.2 M glutamine, 50,000 U L^−1^ penicillin, and 50 mg L^−1^ streptomycin). The hybridomas were selectively cultured in complete medium supplemented with 1% (v/v) HAT for approximately 2 weeks and the supernatants were screened by ELISAs described below. The resulting mAbs were generated by inoculating selected hybridoma cells into Balb/c mice treated with mineral oil. Anti-artemether mAbs were purified from ascite fluids by ammonium sulfate precipitation.

### Indirect Competitive ELISA and Indirect ELISA

The protocol for indirect competitive ELISA (icELISA) and indirect ELISA (iELISA) was the same as that described previously [16], [17]. Monitoring of the titer of antisera, supernatants, or mAbs and screening of positive hybridoma clones were done by iELISA. A microtiter well was coated with hapten-BSA (100 µL per well in 0.05 M carbonate buffer, pH 9.6) for 3 h at 37°C. The plate was washed 3 times with PBST (PBS containing 0.1% (v/v) Tween-20); 100 µL per well of antisera, supernatant, or mAbs both diluted in PBSTG (PBST containing 0.5% (w/v) gelatin, PBSTG) were pipetted and then incubated at 37°C for 30 min. The plate was washed 3 times with PBST. Peroxidase-labeled goat antimouse IgG diluted in PBSTG was then added at 100 µL per well. After being incubated for 30 min at 37°C, the plate was washed 3 times again with PBST; then substrate solution (0.01 M citrate-phosphate buffer, pH 5.5, containing 2 mg mL^−1^ of OPD and 0.04% (v/v) H_2_O_2_) was added at 100 µL per well. The reaction was terminated by adding 50 µL of 2 M H_2_SO_4_ per well. Absorbance was read at 492 nm on a microplate reader.

The specificity of the mAbs was evaluated for cross reactivity with artemisinin derivatives, quinine, primaquine phosphate, chloroquine diphosphate salt pyrimethamine and lumefantrine utilizing icELISA. The procedure of icELISA was generally the same as the iELISA described above, except that the step of 100 µL per well of antisera, supernatant, or mAbs diluted in PBSTG was replaced with 50 µL per well of standard or analytes and 50 µL per well of antisera, supernatant, or mAbs.

### Sample Extraction

The artemether injection (0.5 mL, 80 mg mL^−1^) was transferred quantitatively into a volumetric flask and diluted to 10 mL using acetonitrile. Tablets of artemether (20 mg/tablet) were pulverised with a pestle, acetonitrile (10 mL) added. Artemether capsule (40 mg/capsule) was diluted to 4 mg mL^−1^ using acetonitrile. The samples were then extracted by ultrasonication (SB5200, Branson, Shanghai, China) for 20 min. The extracts were centrifuged at 5000 rpm for 10 min. The supernatants were collected as the final extract and kept at −20°C until ELISA and HPLC analyses.

### HPLC Analysis of Artemether

Standards and artemether samples were analyzed by HPLC according to the procedure of Zhao and Zeng [18]. The HPLC system consisted of a Waters 600E multisolvent delivery system and a Waters 2487 dual λ absorbance detector (Milford, MA, USA). The mobile phase, standards, and sample extracts obtained above were filtered through a 0.45-µm filter prior to HPLC. A C18 reverse-phase column (250×4.6 mm, 5 µm particle size, Thermo, Vantaa, Finland) was used to separate artemether. The mobile phase consisted of acetonitrile and 0.5% acetic acid (70/30, v/v) at a flow rate of 1 mL min^−1^. UV absorption was detected at 210 nm. Artemether standards were prepared in acetonitrile. Calibration curves were constructed in concentrations of 1.0, 2.0, 3.0, 4.0 and 5.0 mg mL^−1^. Artemether solutions were prepared at a concentration of 2 mg mL^−1^. All data were collected and analyzed by using the Waters Millennium^32^ software.

### Data Analysis

The data of icELISA and HPLC were analyzed by paired t-test and Bland-Altman method.

## Results

### Preparation of mAbs against Artemether

To prepare specific mAbs against artemether, 9-O-succinylartemether was conjugated to OVA and used to immunize mice. The antisera collected from the mice after the fourth immunization were screened against the coating antigen (BSA-hapten conjugates). The titer of the antibody was defined as the fold dilution giving an absorbance of 1.0 in iELISA. The mouse with the highest titer and the best percentage inhibition which was evaluated by icELISA was used for further study. Three positive hybridomas were cloned twice by limiting dilution. Two positive clones secreted mAbs against artemether were designated as 2G12E1 and 4H10C9, respectively. In icELISA, mAb 2G12E1 had a lower 50% of inhibition (IC_50_) value of competitive binding to artemether than 4H10C9 (data not shown). Subsequently, 2G12E1 was expanded and used to produce ascites.

### Characterization of the mAb

The cross reactivity of the mAb 2G12E1 was tested using artesunate, dihydroartemisinin, artemisinin and other major antimalarial drugs in icELISA (Table 1). The cross reactivities of dihydroartemisinin and artemisinin were approximately 1.3%, and 2.3%. No competitive inhibition was observed for up to 20,000 ng mL^−1^ of artesunate, quinine, primaquine phosphate, chloroquine diphosphate salt, pyrimethamine, or lumefantrine.

**Table 1:** Cross reactivities of the icELISA with commonly used antimalarial drugs.

Analytes	IC_50_ (ng/mL)	Cross reactivity^a^ (%)
Artemether	3.70±0.14^b^	100±3.9
Dihydroartemisinin	283±4	1.3±0.0
Artemisinin	159±5	2.3±0.1
Artesunate	NI^c^	0
Quinine	NI^c^	0
Primaquine phosphate	NI^c^	0
Chloroquine diphosphate salt	NI^c^	0
Pyrimethamine	NI^c^	0
Lumefantrine	NI^c^	0

a Cross-reactivity (%) = (IC_50_ of artemether/IC_50_ of other compound)×100.

b Data represent means of triplicate ± SD.

c No inhibitions were observed up to 20,000 ng mL^−1^ of the analytes.

### Competitive Inhibition

**Figure 3:**
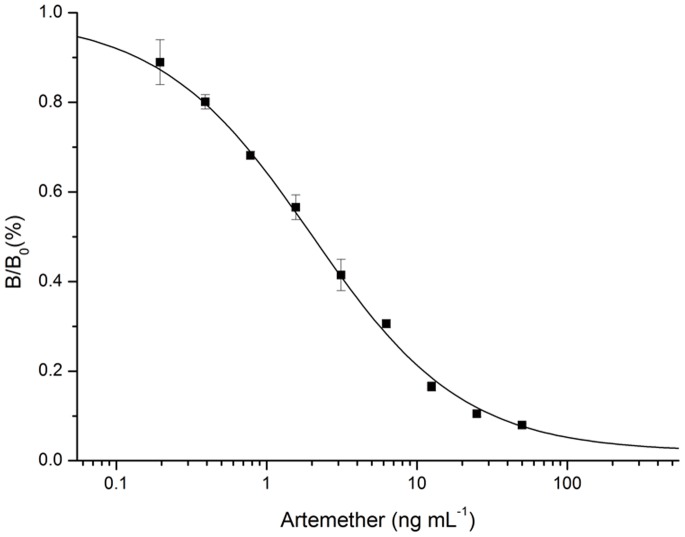
Standard inhibition curve of artemether in icELISA format. B_0_ and B are absorbance in the absence and presence of competitors, respectively. Concentration causing 50% inhibition by artemether was 3.70 ng mL^−1^. Each value represents the mean of three replicates.

The optimal concentrations of coating antigen, mAb, and peroxidase-labeled goat anti-mouse IgG were screened by checkerboard titration. A standard inhibition curve for artemether was established by icELISA under the optimized conditions (Fig. 3). The IC_50_ value and the working range based on 20 to 80% of inhibition were 3.7 ng mL^−1^ and 0.7–19 ng mL^−1^, respectively.

### Comparative Analysis of Artemether Samples with icELISA and HPLC

**Figure 4:**
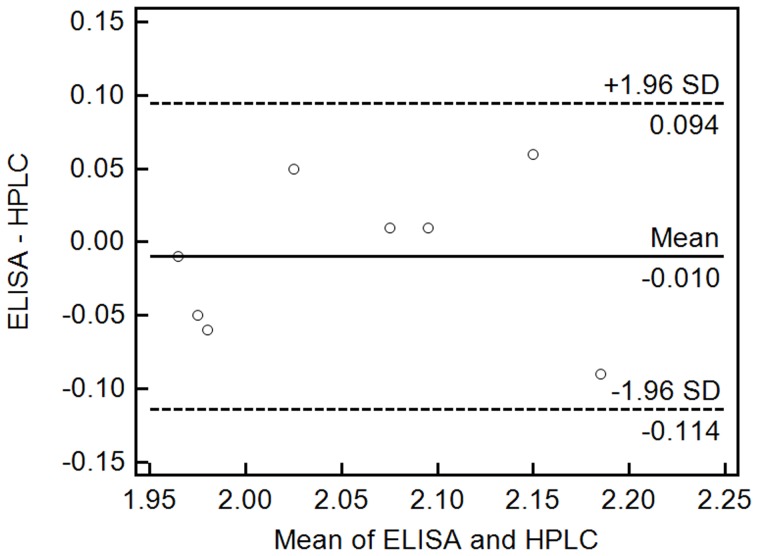
Bland-Altman bias plots for ELISA and HPLC. Quantitating artemether drugs concentration expressed as mg mL^−1^. The solid line represents the bias between the assays, and the dashed lines represent the bias ±1.96-s limits.

The artemether content in eight commercial drug samples was determined by using icELISA and HPLC (Table 1 and Table 2). The results of the icELISA and HPLC were compared by the paired t-test for accuracy at 95% confidence level for seven degrees of freedom (T = −0.545, P = 0.603>0.05), which indicate no significant difference between the two methods in terms of accuracy. Data from these two assays were further analyzed by Bland-Altman bias plot combined with calculation of bias and 95% limits of agreement with 95% confidence intervals (Fig. 4). The mean bias ±1.96 standard deviations were between −0.114 and 0.094 ng mL^−1^. Thus, both statistical procedures suggested that the results from the two methods were highly comparable and the icELISA method could be used for accurate quantification of artemether.

**Table 2:** Comparison of the icELISA and HPLC method for quantitation of artemether in commercial drugs.

Drug^a^	Batch No.	ELISA	HPLC
Artemether injection (Kunming Pharma. Corp.)	99125822	1.96±0.02^b^	1.97±0.08^b^
	20000355.29	1.95±0.12	2.01±0.07
	20011052.01	2.08±0.06	2.07±0.05
	07CM01	2.05±0.01	2.00±0.03
	10 ML02	2.14±0.10	2.23±0.01
Artemether soft capsules (Chongqing Holley Healthpro Pharmaceutical Co., Ltd.)	20110301	2.18±0.11	2.12±0.04
Coartem 20/120 (Beijing Novartis Pharma Ltd.)	X1475	2.10±0.01	2.09±0.03
Co-Falcinum (Cipla Ltd.)	B/NK 01885	1.95±0.14	2.00±0.06

a Each sample was analyzed in triplicate.

b Data represent mean ± SD. The unit for ELISA and HPLC data was mg mL^−1^. The theoretical content of all drugs was 2 mg mL^−1^.

## Discussion

To produce antibodies against artemether, which are too small to be immunogenic, it must be conjugated to a large carrier protein, which functions as the primary immunogen. Among artemisinin and its major derivatives used in antimalarial medicines, only artesunate can be used directly as the hapten for conjugation because the presence of the active succinyl group on position 12 of artemisinin. However, antibodies produced towards the artesunate-carrier protein conjugate showed broad crossactivities [13], [16]. No active groups such as hydroxyl, carbonyl or carboxylic acid on the molecular of artemether can be used to conjugate the carrier protein as immunogen. There are two ways to introduce an active functional group at position 9. One is chemical derivitization, while another is biotransformation. A challenge for the chemical derivitization is the possible breakdown of the peroxide bridge and thus loss of structural similarity between the hapten and the target analyte. Artemether can be readily bio-transformed by *C. elegans* to produce 9-hydroxyartemether [15] and *Streptomyces griseus* (ATCC 13273) to produce artemisitone-9 [19]. 9-Hydroxyartemether can readily react with succinic anhydride to yield 9-O-succinylartemether.

Hapten structure is the basis for antibody’s specific recognition. In general, there are some correlations between the position in the hapten molecule used for conjugation to a carrier protein and the recognition of epitopes on the hapten by the prepared antibodies. The epitopes distant from the site of conjugation tend to be well recognized by antibodies, whereas epitopes neighboring the coupling site tend to be less well recognized. For example, antibodies to abscisic acid (ABA) conjugated to a protein through the C1-carboxyl can recognize ABA methyl ester well than free ABA, while the antibodies to ABA conjugated to the carry protein through the C4’-carbonyl only recognize free ABA [20]. Artemisinin derivatives differ in their structures at position 12 and the other part of the molecules is the same. The reported antibodies to artemisinin derivatives have been all prepared with immunogens on which the haptens were conjugated through the position 12, but cannot discriminate the difference of the derivatives. The mAbs reported by He [16] recognized artesunate, dihydroartemisinin, artemether with cross reactivity of 650%, 57%, 3%, respectively. The mAbs generated by Tanaka et al. [13] showed cross reactivities, which were 630% for artesunate and 30% for dihydroartemisinin, while the artemether polyclonal Abs prepared by Song et al. [21] recognized artesunate, dihydroartemisinin, and artemether equally well. The mAbs raised by artelinic acid-BSA conjugate bound artemisinin and artemether approximately the same [14]. As compared to those antibodies, the mAb 2G12E1 that was prepared by conjugation of the molecule to the carrier protein through position 9 showed high specificity for artemether. The cross reactivities with dihydroartemisinin and artemisinin were approximately 1.3% and 2.3%. No competitive inhibition was observed up to 20,000 ng mL^−1^ of artesunate, quinine, primaquine phosphate, chloroquine diphosphate salt, pyrimethamine or lumefantrine. It means the quantitation of artemether drugs by the mAb 2G12E1-based ELISA will not be greatly influenced by other antimalarial drugs.

The mAb 2G12E1-based ELISA developed in the present study was compared with the previously developed mAb 3H2-based ELISA [16] for the analysis of the active ingredients dihydroartemisinin, artesunate and artemether in commercial ACT drugs. After 200,000 folds dilution of the samples according to the IC_50_ value of the two assays, the artemether drugs were still detected with the 2G12E1-based ELISA, whereas dihydroartemisinin and artesunate drugs gave negative results. In contrast, these three drugs were positively detected with the 3H2-based ELISA. The results indicate that both ELISAs are valuable for quality control of artemisinin-based antimalarial drugs. The most notable advantage of the 2G12E1-based ELISA is its high specificity to artemether only, which allows specific detection and quantitation of artemether in unknown samples.

We further evaluated the icELISA for the analysis of artemether drugs and compared it with the gold standard HPLC. The icELISA had a working range of 0.7–19 ng mL^−1^ for artemether. The limit of detection by the icELISA was approximately 1000 times lower than the HPLC method [4]. The high assay sensitivity can afford up to 100,000- to 1,000,000-fold dilutions of the drugs to completely eliminate matrix interference and thus increase the assay accuracy. The results obtained from the icELISA agreed well with those from HPLC analysis. Compared with reported artemether instrumental methods, TLC and immunoassays, the newly developed ELISA is selective, sensitive and allows large sample throughput. MAb 2G12E1 can be a valuable reagent to prepare artemether-specific strip assays. From a practical point of view, the specific mAb 2G12E1 and its simple and economical assays have a good potential for artemether drug quality control in field settings of malaria endemic areas.
